# Exploring the origins of nucleation

**DOI:** 10.7554/eLife.67269

**Published:** 2021-03-10

**Authors:** Katarzyna Marta Zoltowska, Lucía Chávez-Gutiérrez

**Affiliations:** 1 VIB-KU Leuven Center for Brain & Disease ResearchLeuvenBelgium; 2 Department of Neurosciences, Leuven Research Institute for Neuroscience and Disease (LIND)LeuvenBelgium

**Keywords:** amyloid, deep mutagenesis, nucleation, aggregation, Alzheimer's, *S. cerevisiae*

## Abstract

An approach called deep mutational scanning is improving our understanding of amyloid beta aggregation.

**Related research article** Seuma M, Faure A, Badia M, Lehner B, Bolognesi B. 2021. The genetic landscape for amyloid beta fibril nucleation accurately discriminates familial Alzheimer’s disease mutations. *eLife* **10**:e63364. doi: 10.7554/eLife.63364

Alzheimer’s disease is a progressive neurodegenerative disease that initially compromises memory and ultimately stops people from being able to perform everyday tasks. The exact causes of the disease are still unknown, but genetic, lifestyle and environmental factors could all play a role, with age being the strongest risk factor for developing the condition. The lack of treatment to halt or slow down the progression Alzheimer’s disease makes it one of the greatest challenges of our times.

Alzheimer’s disease begins decades before the appearance of clinical symptoms. It is thought that changes in the metabolism of a peptide called amyloid beta (Aβ) lead to its misfolding. Although the details are not fully understood, scientists propose that the accumulation of these misfolded molecules in the brain triggers a series of molecular events which, in turn, lead to neuroinflammation, the aggregation of tau proteins in neurons, and eventually, neuronal death (Selkoe and Hardy, 2016).

Preventing the build-up of Aβ has been explored as a potential therapeutic approach. However, designing strategies that specifically target the formation of neurotoxic Aβ, rather than the aggregation of Aβ in general, requires a detailed understanding (in terms of both mechanisms and kinetics) of how the protein misfolds and how this is connected to the disease (Yang et al., 2017; Brinkmalm et al., 2019). Now, in eLife, Benedetta Bolognesi (IBEC in Barcelona), Ben Lehner (the Center for Genomic Regulation, also in Barcelona) and colleagues – including Mireia Seuma as first author, Andre Faure and Marta Badia – report new insights into how mutations affect the initial nucleation of Aβ aggregates (Seuma et al., 2021).

The researchers used an approach called deep mutational scanning (Gray et al., 2019) to generate a library of 468 single and 14,015 double mutant Aβ variants to see how the mutations affect the ability of Aβ to form new aggregates. They focused on the first step of this process, known as nucleation, and then determined the relationship between amino acid sequence and the nucleation rate of Aβ42 (a form of Aβ that contains 42 amino acids) (Figure 1).

**Figure 1.**
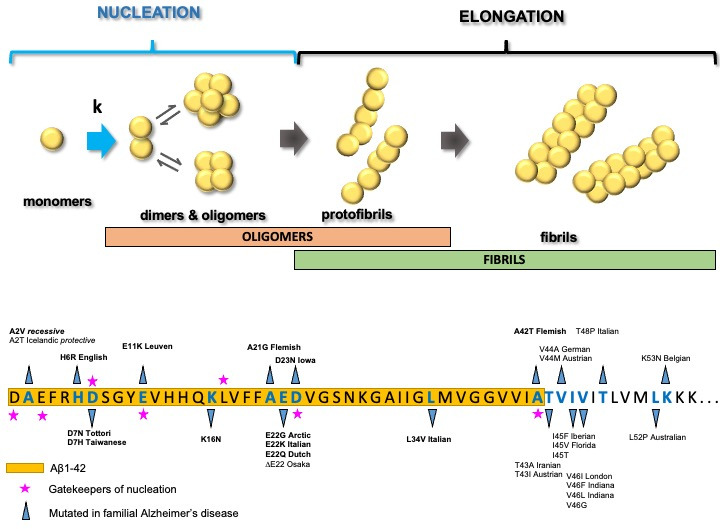
Amyloid beta peptide and Alzheimer’s disease. It is thought that Alzheimer’s disease is caused by amyloid beta (Aβ) (yellow circles; top) first forming dimers and then oligomers in a process called nucleation, with the oligomers then going on to build protofibrils and fibrils. Aggregated Aβ then deposits in amyloid plaques, which are a pathological hallmark of Alzheimer’s disease. Familial Alzheimer’s disease is hereditary and is marked by an unusually early onset of symptoms. Several genes have been linked to this form of the disease, including the gene for the amyloid precursor protein (*APP*). Certain amino acid residues in Aβ are called 'gatekeepers’ of nucleation (magenta stars; bottom) because they prevent nucleation. The mutations indicated (blue arrows), affecting amino acid residues highlighted in blue, cause familial Alzheimer’s disease or protect (A2T) against it. Pathogenic mutations in APP are shown in text (the numbering corresponds to the positions with Aβ). The sequence of amino acids shown here is from the N-terminal of Aβ42. Seuma et al. found that dominant pathogenic mutations within the Aβ42 peptide (indicated in bold text; bottom) all display increased rate of nucleation.

Seuma et al. found that mutations that reduce nucleation are clustered in the hydrophobic part of Aβ42 (the C-terminal), while those that increase this process are located in the N-terminal part, which is hydrophilic. This suggests that Aβ is organized in a modular manner, with the N- and C-terminal parts having different roles in nucleation and aggregation. Intriguingly, such a modularity is also reflected in the distribution of pathogenic mutations in the Aβ precursor called APP (Figure 1). These mutations have been linked to familial Alzheimer’s disease, a rare genetic form presenting a much earlier onset (people usually develop symptoms in their thirties, forties and fifties).

Seuma et al. further identified five negatively charged residues in Aβ42, which acted as 'gatekeepers’, preventing the peptides from sticking to each other (Rousseau et al., 2006). Mutations at these sites frequently resulted in increased nucleation. This raises several questions: could the gatekeepers of nucleation prevent neurodegeneration; and do mutations that promote nucleation prime the system for the onset of Alzheimer’s disease?

To investigate this further, Seuma et al. studied the nucleation rates for 12 mutations in Aβ42, which have been linked to familial Alzheimer’s disease. The mutations all resulted in increased nucleation rates, although the rates did not correlate with the severity of the disease (as reflected by the age at disease onset). These results suggest that Aβ nucleation plays a key role in the formation of neurotoxic aggregates, but other factors are also involved.

It is worth noting that the reservoir of Aβ in the brain contains a heterogeneous mixture of peptides of varying lengths, generated by the sequential cleavage of APP. Pathogenic mutations in both APP and the enzymes responsible for its cleavage favor the production of longer Aβ peptides (Szaruga et al., 2017). Importantly a large number of mutations linked to familial Alzheimer’s disease do not change the amino acid sequence of Aβ42, but affect the composition of Aβ profiles by shifting them towards longer peptides (Szaruga et al., 2015). Whether these pathogenic changes in peptide length (and hydrophobicity) lead to an increase in nucleation, and how this is related to the clinical phenotypes of Alzheimer’s disease, warrant further investigation.

Disentangling the complex pathogenesis of Alzheimer’s disease will likely require the interrogation of various pathogenic variants under controlled conditions and the plotting of biochemical and cellular data against clinical severity of the condition. The study by Seuma et al. certainly shows that the nucleation assay is a valuable tool for such investigations.
